# Supplementary material for: DNA viral community enhances microbial carbon fixation capacity via auxiliary metabolic genes in contaminated soils
Source: Nat Commun. 2025 Nov 13;16:9984. doi: 10.1038/s41467-025-64938-2 (PMC12615777; doi:10.1038/s41467-025-64938-2)
Supplement: Supplementary file 1 — Supplementary Information [file 41467_2025_64938_MOESM1_ESM.pdf]

DNA viral community enhances microbial carbon fixation capacity via auxiliary metabolic genes in contaminated soils

Author list

Jia-nan Lu[1], Yuanqing Chao[1*], Li Tian[1], Xi Zhong[1], Ziwu Chen[1], Huan He[2], Bi Huang[3], Mengyao Li[1], Zekai Feng[1], Huayuan Feng[1], Chang Hu[1], Shunkang Zhou[2], Liqi Zhang[4], Yulu Yang[1], Zhepu Ruan[2], Kengbo Ding[1], Ying Yang[4], Ke Yuan[4], Wenshen Liu[1], Hua Qi[2], Yue Cao[1], Ying-heng Fei[5], Ning Ling[6], Shizhong Wang[1], Ye-tao Tang[1], Tiangang Luan[3], Zhihong Xu[7], Rongliang Qiu[1,2*]

Affiliations

1 School of Environmental Science and Engineering, Guangdong Provincial Key Laboratory of Environmental Pollution Control and Remediation Technology, Sun Yat-sen University, Guangzhou, 510006, China

2 Guangdong Provincial Key Laboratory of Agricultural & Rural Pollution Abatement and Environmental Safety, College of Natural Resources and Environment, South China Agricultural University, Guangzhou, 510642, China

3 State Key Laboratory of Biocontrol, School of Life Sciences, Sun Yat-sen University, Guangzhou, 510275, China

4 School of Marine Sciences, Sun Yat-Sen University, Zhuhai, 519082, China

5 School of Environmental Science and Engineering, Guangzhou University, Guangzhou 510006, China

6 State Key Laboratory of Herbage Improvement and Grassland Agro-Ecosystems, College of Pastoral Agriculture Science and Technology, Lanzhou University, Lanzhou 730020, China

7 School of Environment and Science - Chemistry and Forensic Science, Griffith University, Brisbane, Queensland, Australia

Corresponding author (asterisk marked): Yuanqing Chao and Rongliang Qiu

Email address: chaoyuanq@mail.sysu.edu.cn.

Email address: qiurl@scau.edu.cn

Supplementary Figures

Supplementary Fig. 1. Rarefaction curve of viral operational taxonomic units (vOTUs).
Supplementary Fig. 2. Quality and lifestyle distribution of viral genomes in contaminated soils.
Supplementary Fig. 3. Quality and lifestyle distribution of viral genomes in non-contaminated soils.
Supplementary Fig. 4. Correlation between virus and prokaryote and viral infection model in contaminated soils.
Supplementary Fig. 5. Host distribution and viral infection model in non-contaminated soils.
Supplementary Fig. 6. Functional annotation of virus and gene analysis of C fixation in contaminated soils.
Supplementary Fig. 7. Phylogenetic trees of C-fixation AMGs.
Supplementary Fig. 8. 3D protein structure parameters of C-fixation AMGs.
Supplementary Fig. 9. AMGs expression, purification, and activity assay.
Supplementary Fig. 10. Results and verification of phage transduction experiments.
Supplementary Fig. 11. Distance decay relationship (DDR) of prokaryotic and viral communities at different scales.
Supplementary Fig. 12. Variation of gene abundance related to C metabolism.

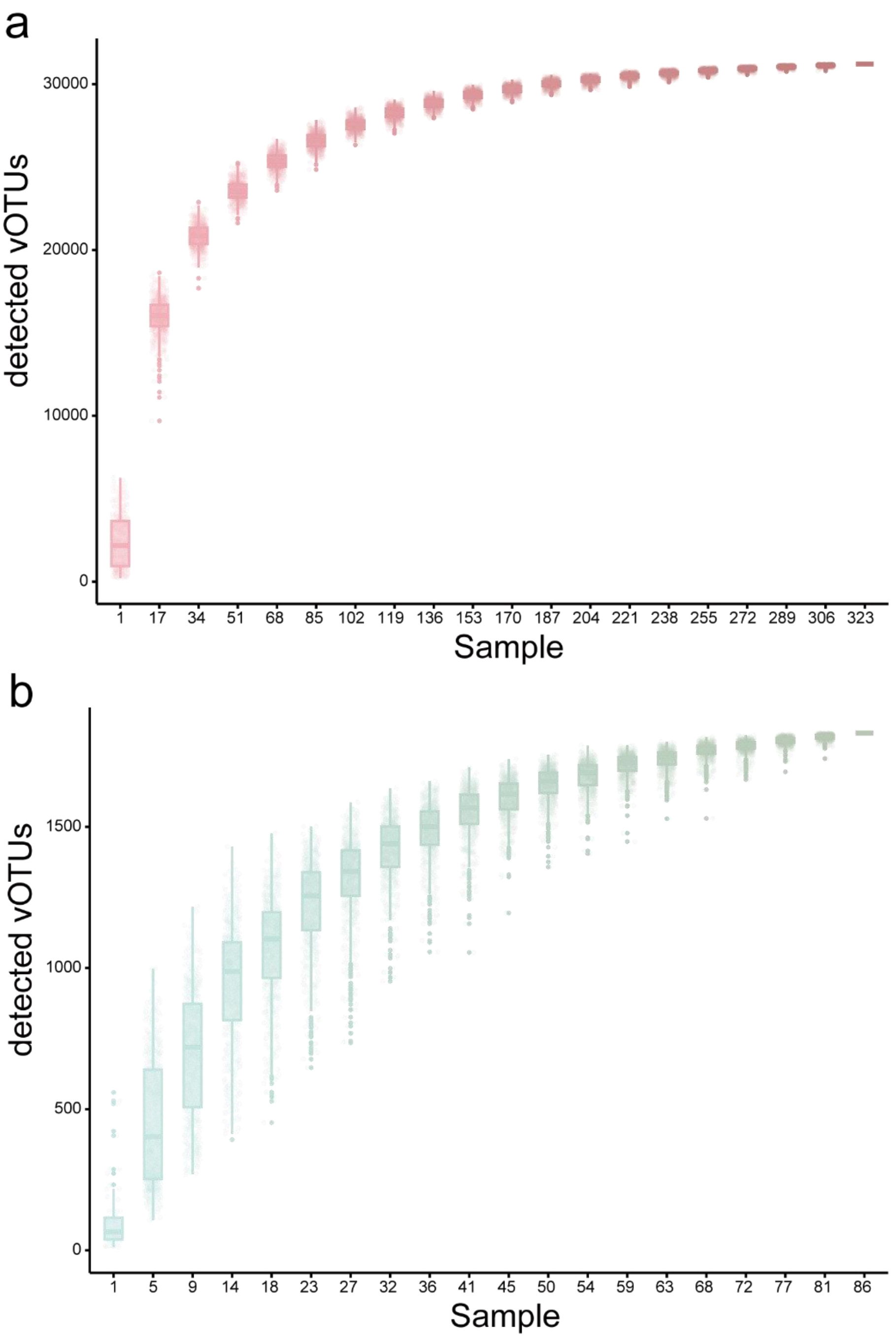

Supplementary Fig. 1 | Rarefaction curve of viral operational taxonomic units (vOTUs). a Rarefaction curve in contaminated soils. **b** Rarefaction curve in uncontaminated soils. Boxes show medians/quartiles. Whiskers extend to the most extreme vales within 1.5 interquartile ranges. Points shown beyond the whiskers are outliers.

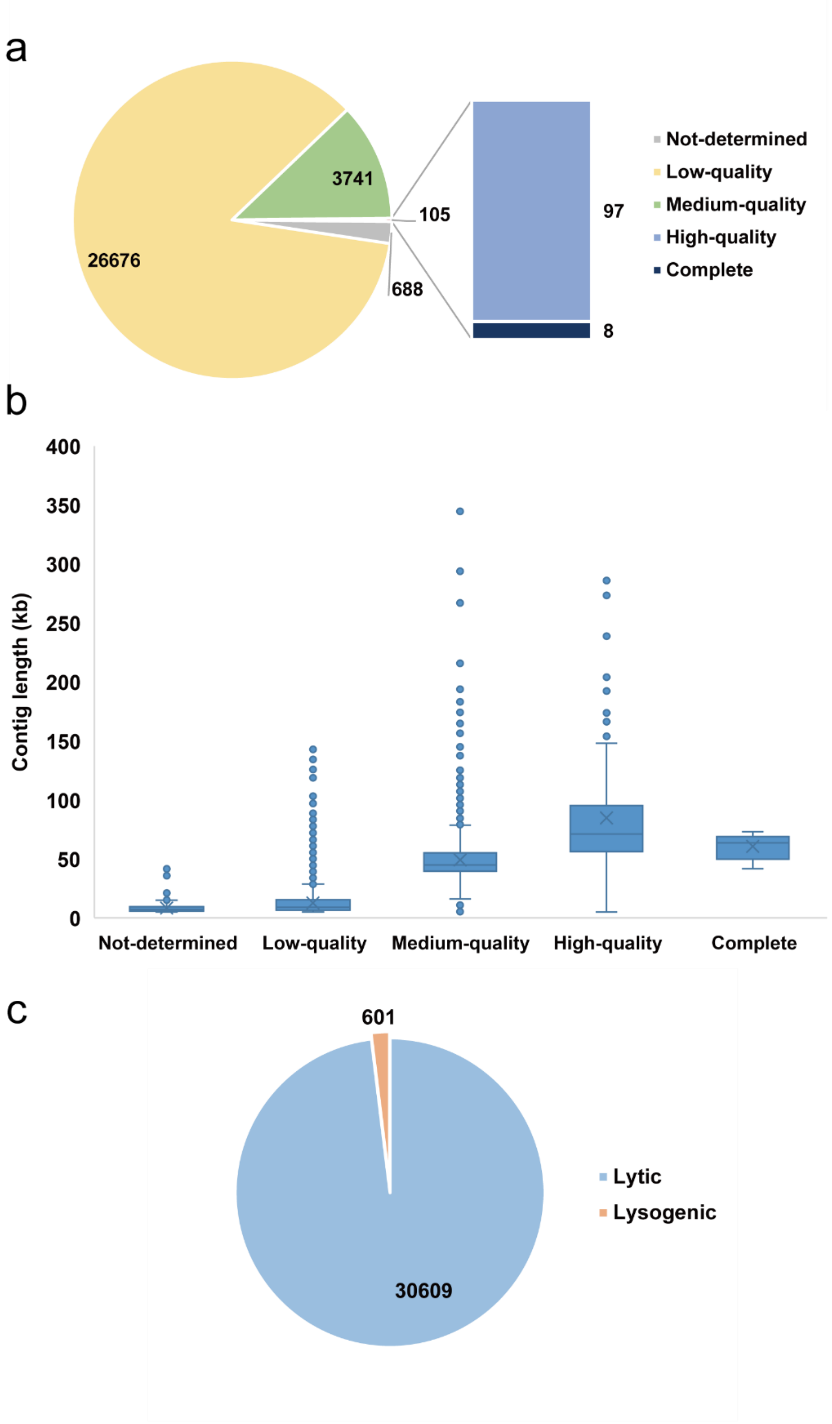

Supplementary Fig. 2 | Quality and lifestyle distribution of viral genomes in contaminated soils. a Quality distribution of viral genomes. **b** Distribution of genome lengths corresponding to different sequence qualities. Boxes show medians/quartiles. Whiskers extend to the most extreme vales within 1.5 interquartile ranges. Points shown beyond the whiskers are outliers. **c** Lifestyles of virus in contaminated soils.

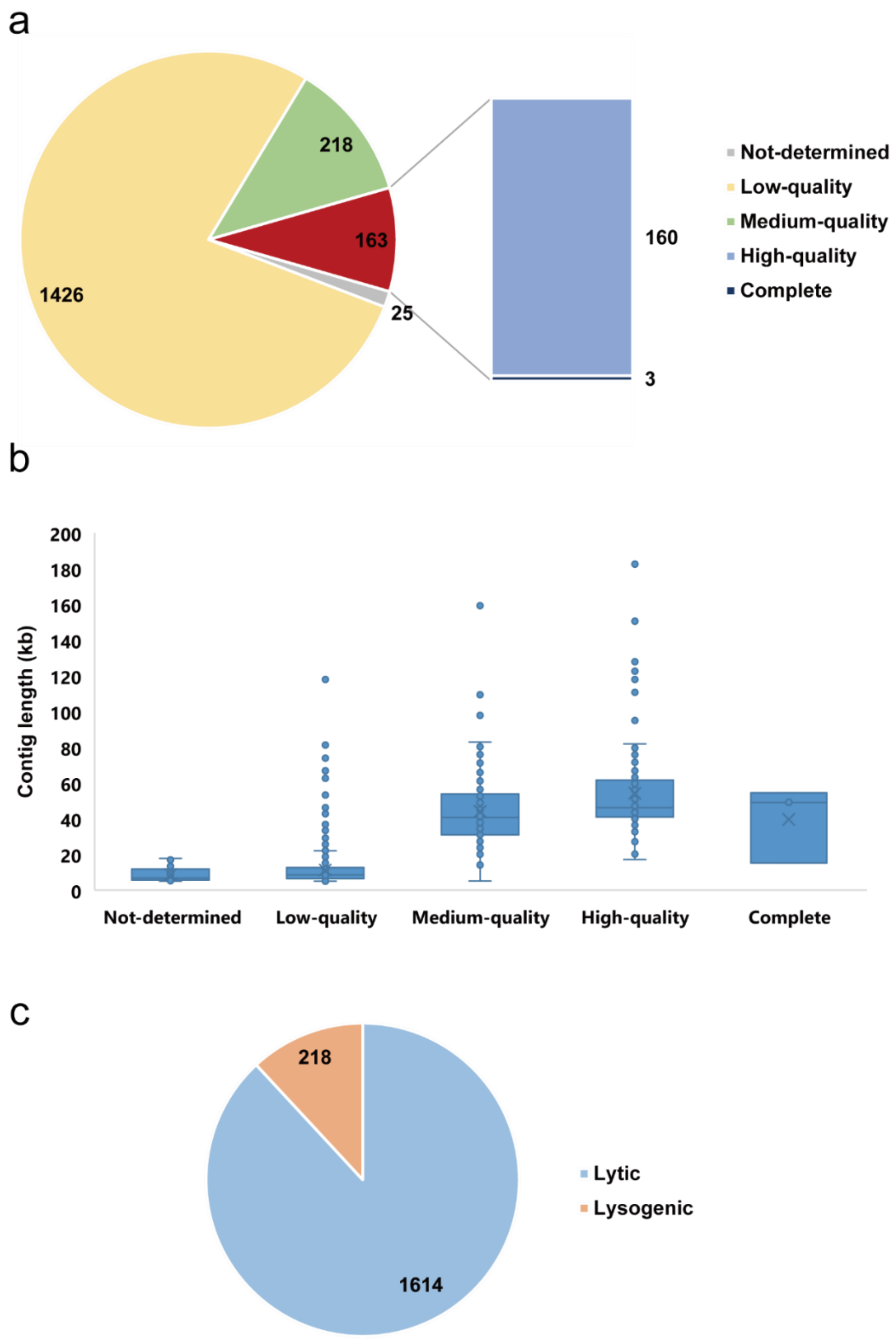

Supplementary Fig. 3 | Quality and lifestyle distribution of viral genomes in non-contaminated soils. a Quality distribution of viral genomes. **b** Distribution of genome lengths corresponding to different sequence qualities. Boxes show medians/quartiles. Whiskers extend to the most extreme vales within 1.5 interquartile ranges. Points shown beyond the whiskers are outliers. **c** Lifestyles of virus in non-contaminated soils.

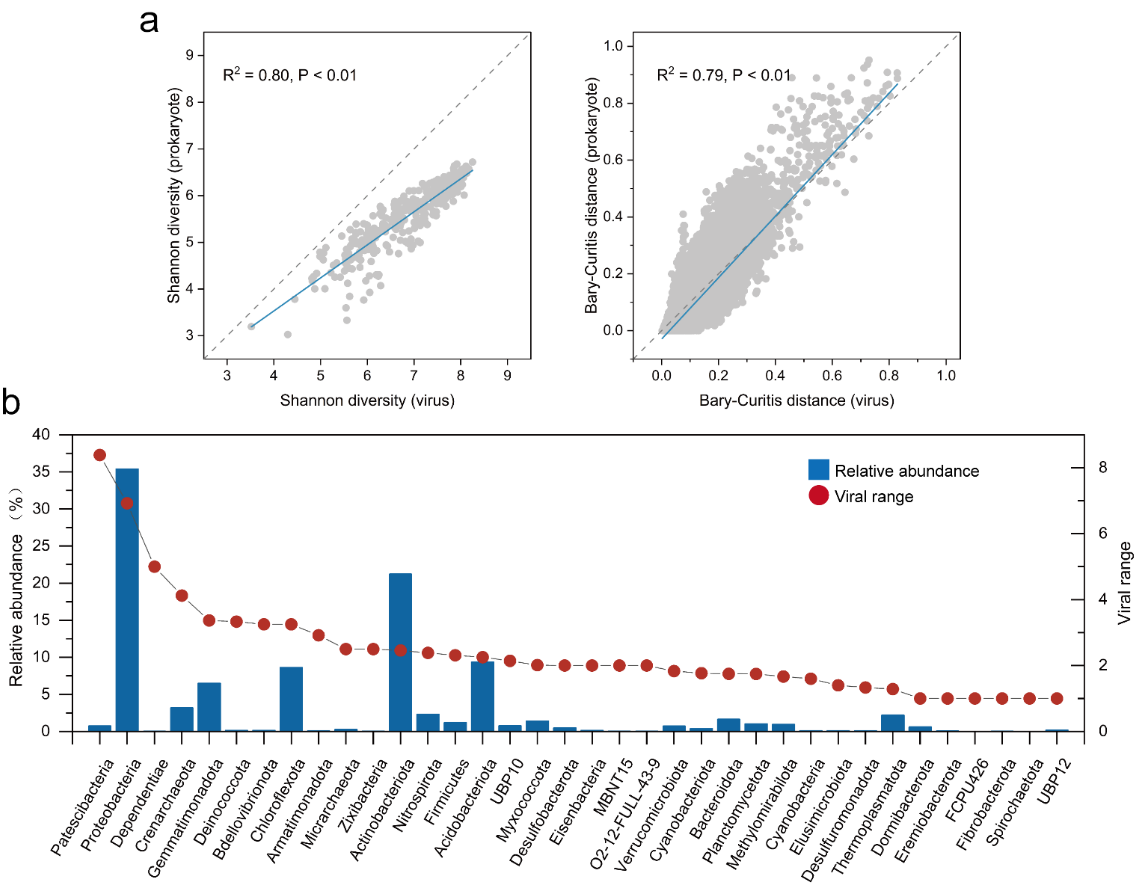

Supplementary Fig. 4 | Correlation between virus and prokaryote and viral infection model in contaminated soils. a Significant Pearson correlation between Shannon diversity and Bary-Curitis distance of viruses and prokaryotes. Statistical significance for regression coefficients was assessed using a two-sided *t*-test (n = 323 samples). **b** Bar graph showed the relative abundance of prokaryotic hosts (blue), while the line chart indicated viral range (circle) by host lineage.

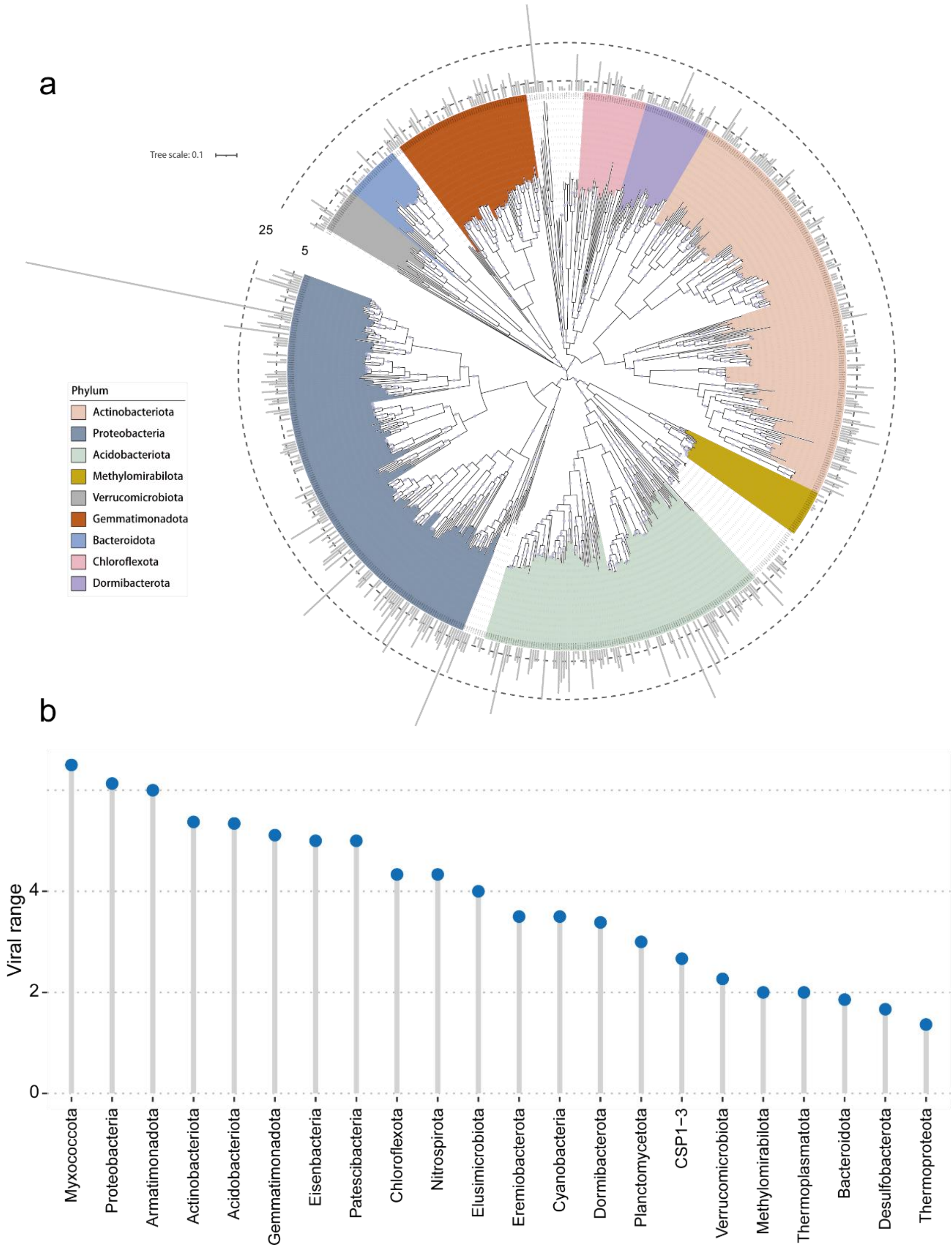

Supplementary Fig. 5 | Host distribution and viral infection model in non-contaminated soils. a Phylogenetic tree and viral population distribution based on MAGs in non-contaminated soils. Major phylum-level bacterial taxa are annotated. The outer bar chart represents the virus association for each MAG. **b** The line chart indicated viral range (circle) by prokaryotic host lineage in non-contaminated soils.

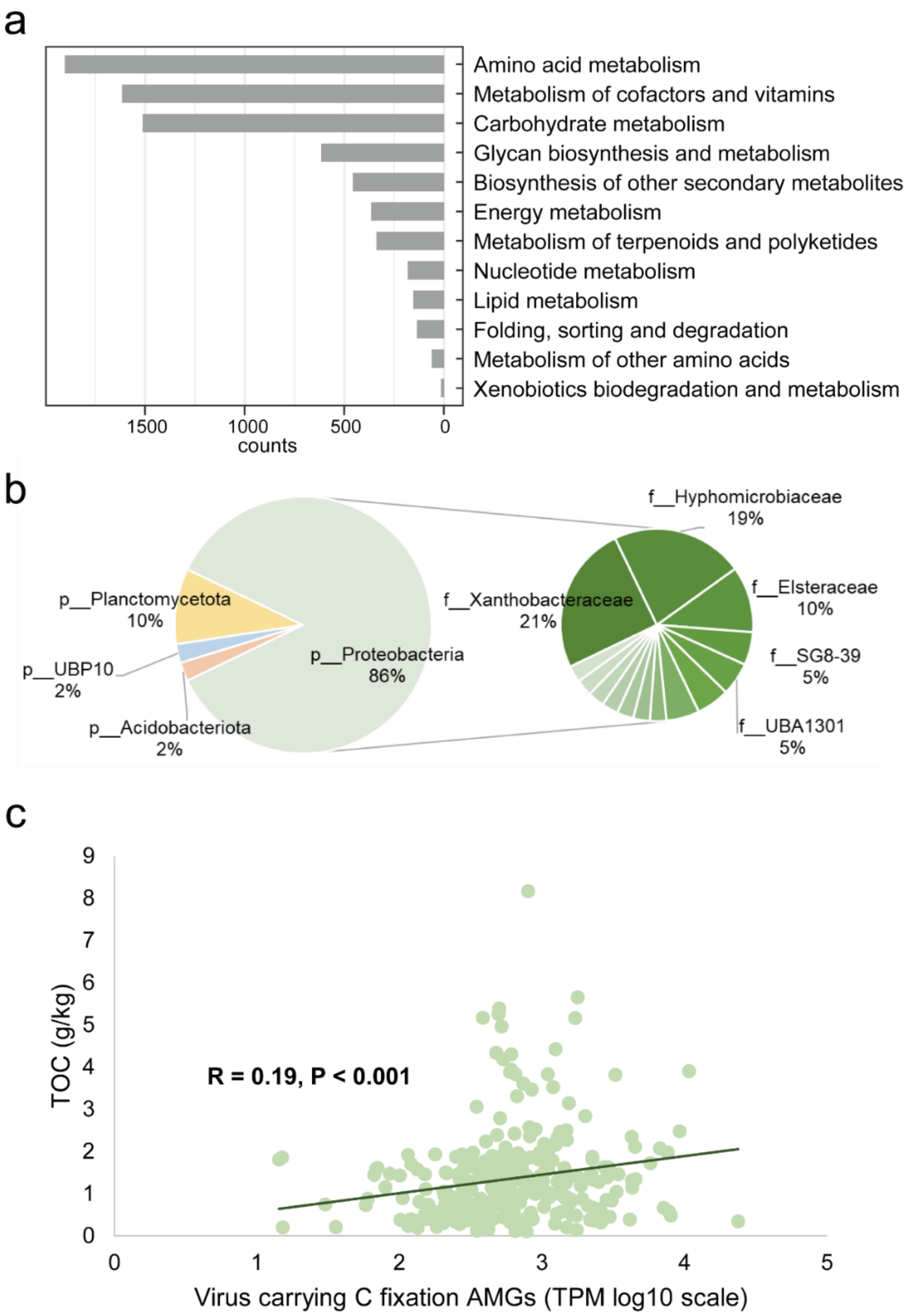

Supplementary Fig. 6 | Functional annotation of virus and gene analysis of C fixation in contaminated soils. a Functional annotation of virus in KEGG. The bar graph represents the distribution of the number of AMGs. **b** host distribution of virus carrying C-fixation AMGs. **c** Correlation analysis of the abundance of virus carrying C-fixation AMGs and TOC. Statistical significance for regression coefficients was assessed using a two-sided *t*-test (n = 323 samples).

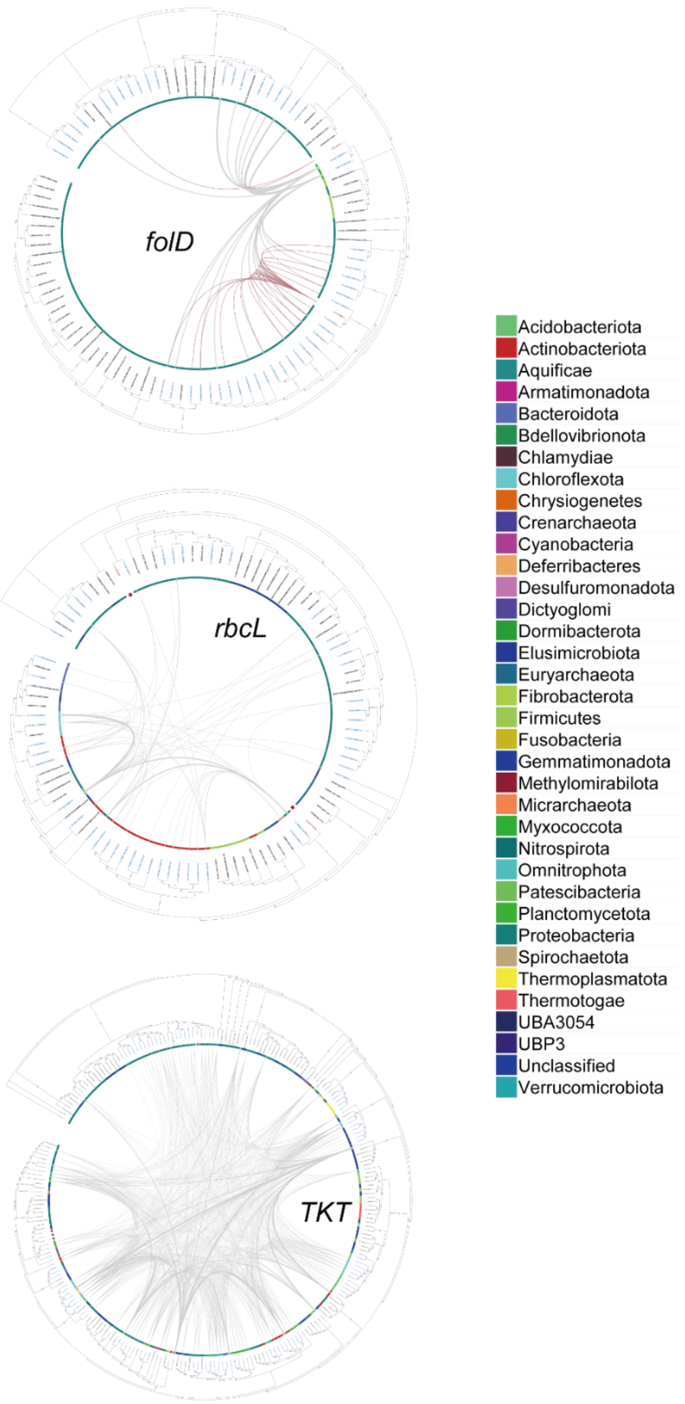

Supplementary Fig. 7 | Phylogenetic trees of C-fixation AMGs. Different lineages of AMGs from bacteria are assigned different range colors according to their taxonomic relationships, as shown in the legend. The red and blue labels represent sequences obtained from mining viruses and MAGs in this study, respectively. The gray lines indicate the predicted horizontal gene transfer events that occurred, and the red lines indicate the exact horizontal gene transfer that occurred between the viral AMGs and MAGs in situ.

rbcL

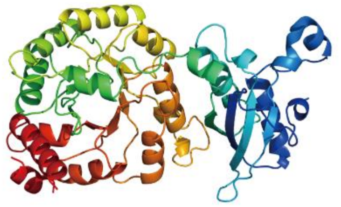

Confidence	**100.0%**
Sequence Identity	**25%**
Query Coverage	**96%**
Protein model organism	*Hyperthermus butylicus DSM 5456*
Expression system	*Escherichia coli BL21(DE3)*

ppdK

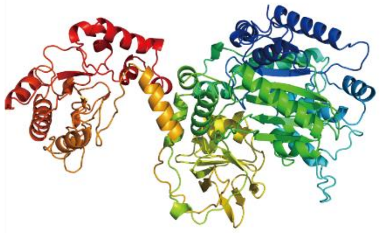

Confidence	**100.0%**
Sequence Identity	**36%**
Query Coverage	**99%**
Protein model organism	*[Clostridium] symbiosum*
Expression system	*Escherichia coli BL21(DE3)*

TKT

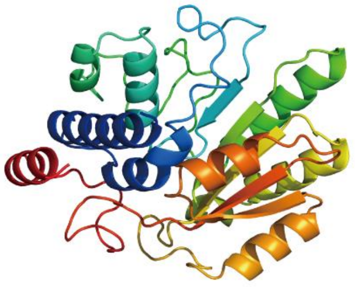

Confidence	**100.0%**
Sequence Identity	**35%**
Query Coverage	**98%**
Protein model organism	*Homo sapiens*
Expression system	*Escherichia coli BL21(DE3)*

Supplementary Fig. 8 | 3D protein structure parameters of C-fixation AMGs.

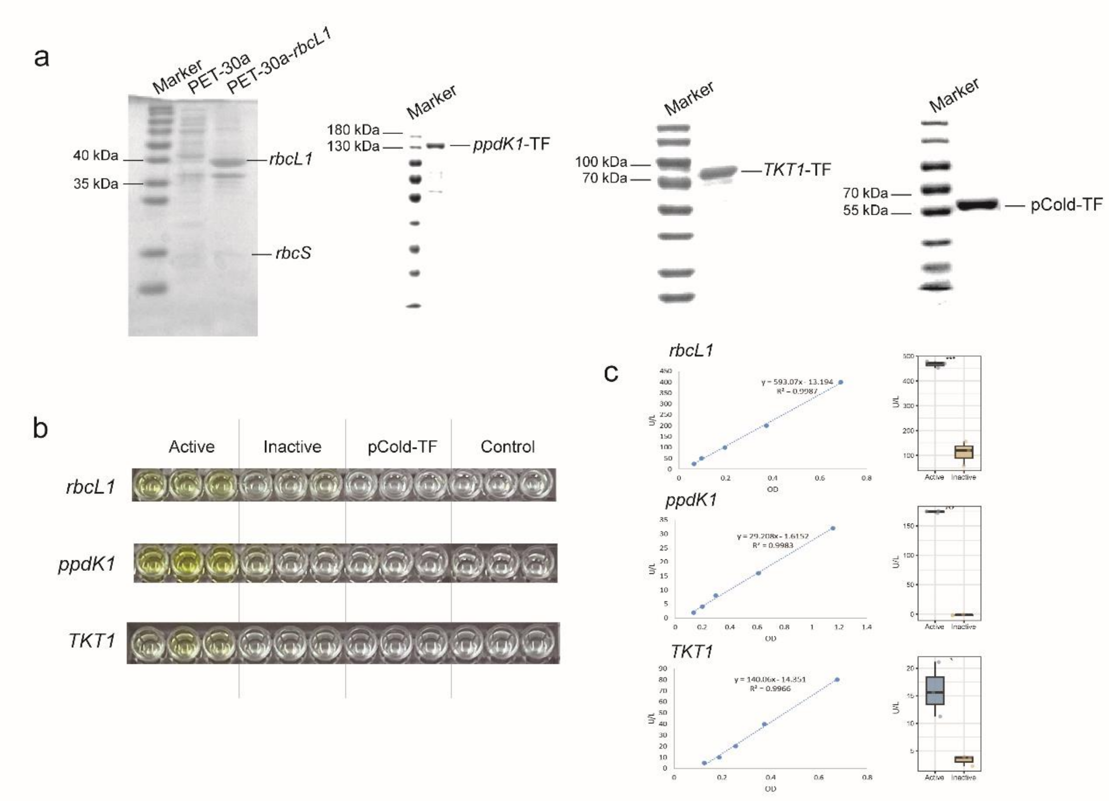

Supplementary Fig. 9 | AMGs expression, purification, and activity assay. a SDS-PAGE of purified vAMGs with the TF-Tag protein. **b** Activity determination of vAMGs. **c** Enzyme activity standard curves of vAMGs and activity comparisons with inactive treatments. Statistical significance for regression coefficients was assessed using a two-sided *t*-test (n = 3 samples). Boxes show medians/quartiles. Whiskers extend to the most extreme vales within 1.5 interquartile ranges. Points shown beyond the whiskers are outliers.

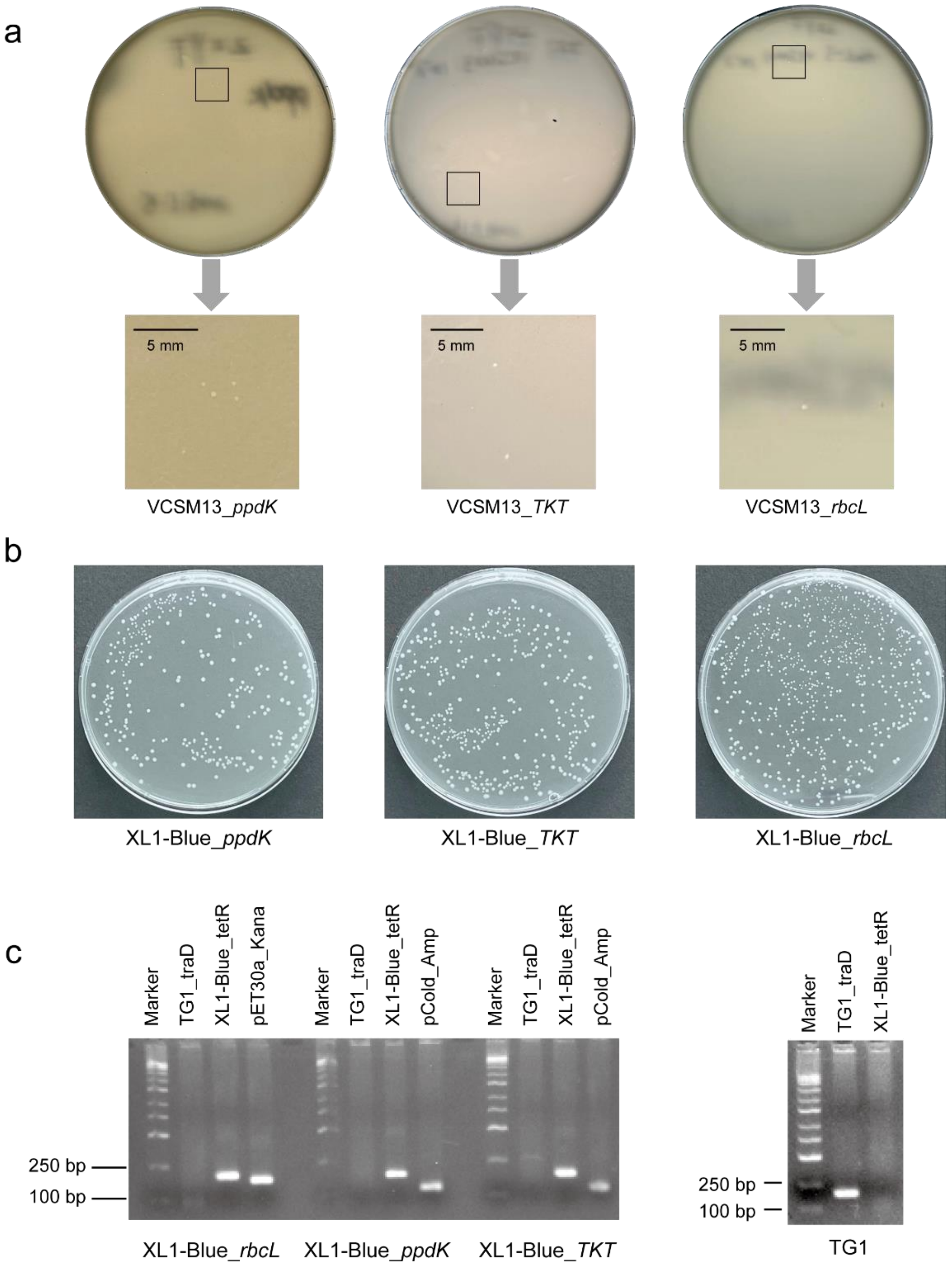

Supplementary Fig. 10 | Results and verification of phage transduction experiments. a Laques formed by phage infection of host cells. **b** Bacterial colonies obtained by antibiotic screening after successful phage transduction. **c** PCR amplification used to verify the exclusion of host contamination.

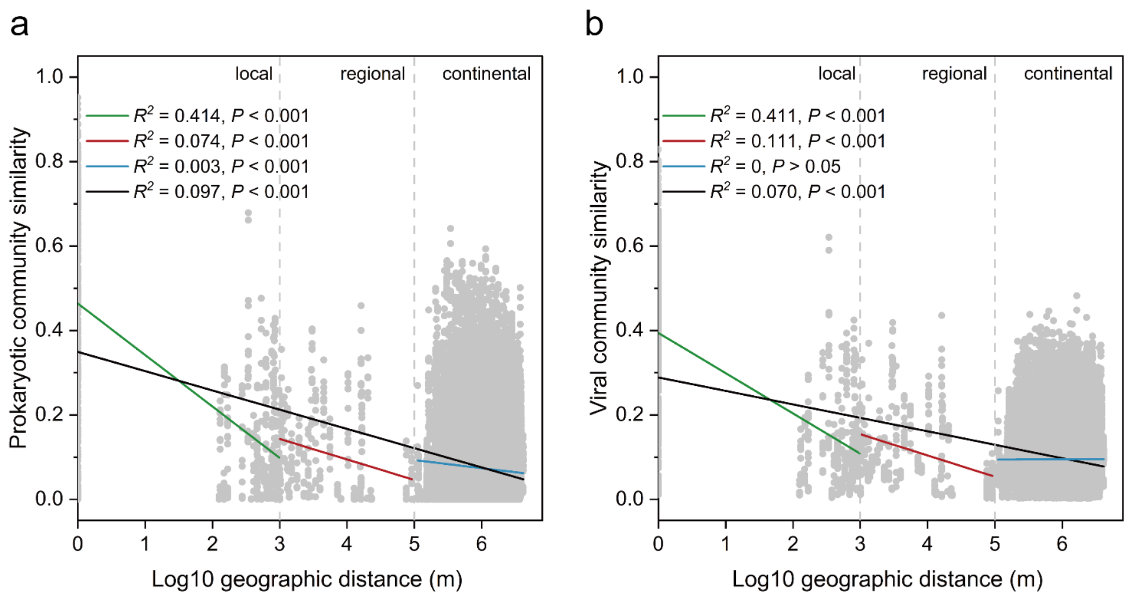

Supplementary Fig. 11 | Distance decay relationship (DDR) of prokaryotic and viral communities at different scales. Statistical significance for regression coefficients was assessed using a two-sided *t*-test (n = 323 samples).

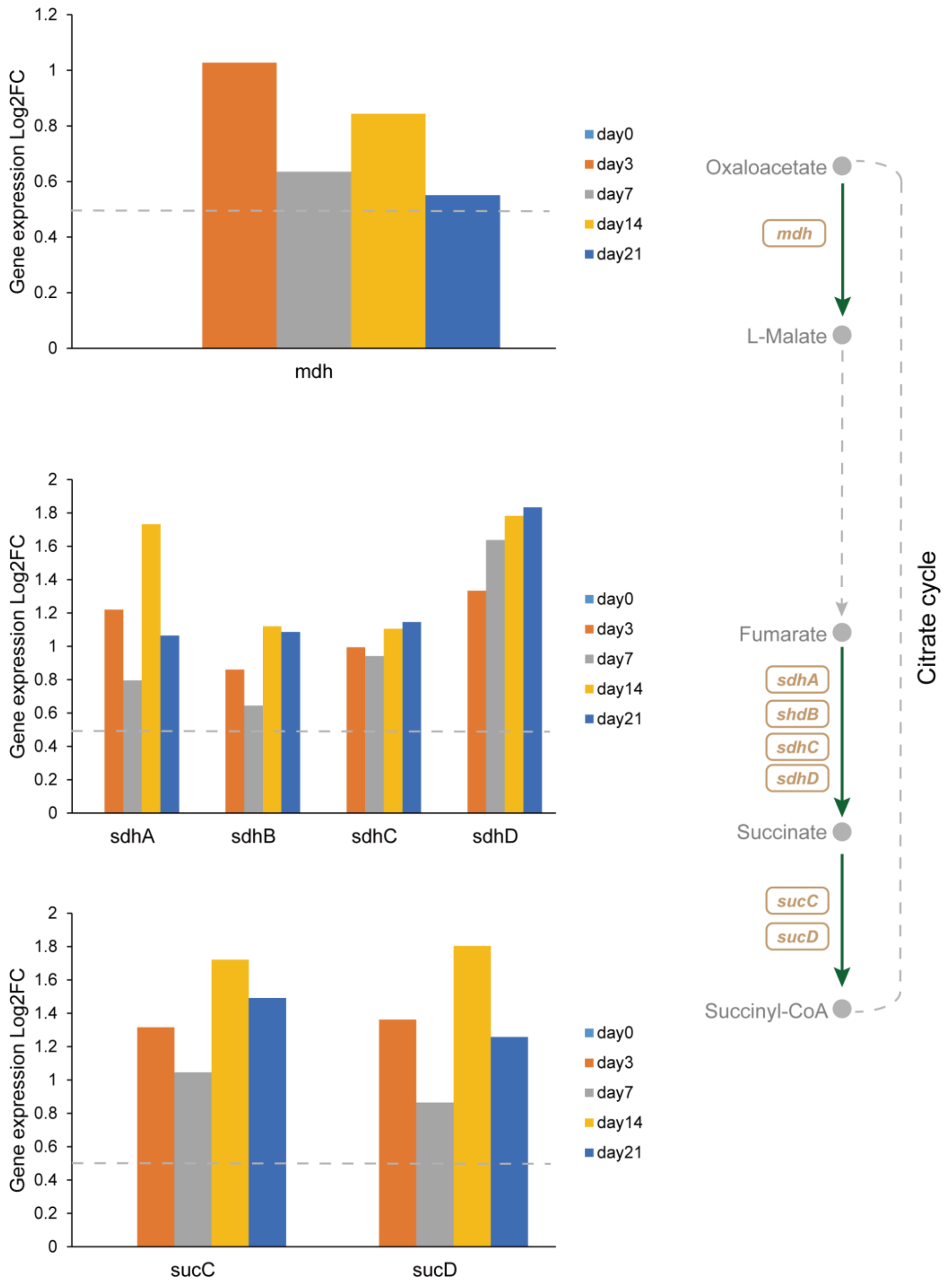

Supplementary Fig. 12 | Variation of gene abundance related to C metabolism.
