# Supplementary material for: DNA viral community enhances microbial carbon fixation capacity via auxiliary metabolic genes in contaminated soils
Source: Nat Commun. 2025 Nov 13;16:9984. doi: 10.1038/s41467-025-64938-2 (PMC12615777; doi:10.1038/s41467-025-64938-2)
Supplement: Supplementary file 2 — Description of Additional Supplementary Files [file 41467_2025_64938_MOESM2_ESM.pdf]

Description of Additional Supplementary Files

File Name: Supplementary Data 1

Description: Detailed information of the sampling sites of this study and selected environmental factors of these sites

File Name: Supplementary Data 2

Description: The species distribution and relative abundance of vOTUs in contaminated soils

File Name: Supplementary Data 3

Description: The species distribution and relative abundance of vOTUs in non-contaminated soils

File Name: Supplementary Data 4

Description: Detailed information of vOTUs identified in contaminated soils

File Name: Supplementary Data 5

Description: Detailed information of vOTUs identified in non-contaminated soils

File Name: Supplementary Data 6

Description: Viral clusters overview with public databases (IMG/VR v4)

File Name: Supplementary Data 7

Description: Shared vOTUs with public databases (IMG/VR v4)

File Name: Supplementary Data 8

Description: Detailed information of MAGs identified in contaminated soils

File Name: Supplementary Data 9

Description: Detailed information of MAGs identified in non-contaminated soils

File Name: Supplementary Data 10

Description: Results of vOTU-host linkages illustrated in contaminated soils

File Name: Supplementary Data 11

Description: Results of vOTU-host linkages illustrated in non-comtaminated soils

File Name: Supplementary Data 12

Description: The functional distribution of vOTUs identified in contaminated soils

File Name: Supplementary Data 13

Description: Genomic information of the C-fixation AMGs

File Name: Supplementary Data 14

Description: Results of variation in abundance (FPKM) of bacterial community after viral inoculation

File Name: Supplementary Data 15

Description: Information of primer sequences for qPCR and RT-qPCR
