# Supplementary material for: DNA viral community enhances microbial carbon fixation capacity via auxiliary metabolic genes in contaminated soils
Source: Nat Commun. 2025 Nov 13;16:9984. doi: 10.1038/s41467-025-64938-2 (PMC12615777; doi:10.1038/s41467-025-64938-2)
Supplement: Supplementary file 4 — Reporting Summary [file 41467_2025_64938_MOESM4_ESM.pdf]

nature portfolio

Corresponding author(s): Yuanqing Chao
Last updated by author(s): Sep 12, 2025

Reporting Summary

Nature Portfolio wishes to improve the reproducibility of the work that we publish. This form provides structure for consistency and transparency in reporting. For further information on Nature Portfolio policies, see our Editorial Policies and the Editorial Policy Checklist.

Statistics

For all statistical analyses, confirm that the following items are present in the figure legend, table legend, main text, or Methods section.

n/a	Confirmed	
☐	☒	The exact sample size (*n*) for each experimental group/condition, given as a discrete number and unit of measurement
☐	☒	A statement on whether measurements were taken from distinct samples or whether the same sample was measured repeatedly
☐	☒	The statistical test(s) used AND whether they are one- or two-sided<br>*Only common tests should be described solely by name; describe more complex techniques in the Methods section.*
☒	☐	A description of all covariates tested
☐	☒	A description of any assumptions or corrections, such as tests of normality and adjustment for multiple comparisons
☐	☒	A full description of the statistical parameters including central tendency (e.g. means) or other basic estimates (e.g. regression coefficient) AND variation (e.g. standard deviation) or associated estimates of uncertainty (e.g. confidence intervals)
☐	☒	For null hypothesis testing, the test statistic (e.g. *F*, *t*, *r*) with confidence intervals, effect sizes, degrees of freedom and *P* value noted<br>*Give P values as exact values whenever suitable.*
☒	☐	For Bayesian analysis, information on the choice of priors and Markov chain Monte Carlo settings
☒	☐	For hierarchical and complex designs, identification of the appropriate level for tests and full reporting of outcomes
☐	☒	Estimates of effect sizes (e.g. Cohen's *d*, Pearson's *r*), indicating how they were calculated

Our web collection on statistics for biologists contains articles on many of the points above.

Software and code

Policy information about availability of computer code

Data collection

Open source tools:
1. Quality control of raw data: Trimmomatic (version 0.35)
2. Assembly of clean reads: MEGAHIT (version 1.2.9)
3. Binning of assembled contigs: MetaWRAP (version 1.3)
4. Clustering and phylogeny of metagenome-assembled Ggenomes: dRep (version 2.6.2) and GTDB-Tk (version 0.2.2)
5. Identification of viral sequences: VirSorter (version 1.0.5) and VIBRANT (version 1.2.1)
6. Quality-checked of viral sequences: CheckV (version 0.6.0)
7. Clustering of viral sequences: CD-HIT-EST (version 4.8.1) and vConTACT (version 2)
8. Taxonomic assignment of vOTUs: PhaGCN (version 2)
9. ORFs predicting: Prodigal (version 2.6.3)
10. Gene function annotation: Diamond (version 0.9.24.125)
11. Reads mapping and quantification: BamM (version 1.7.3) and CoverM (version 0.3.1)
12. Host prediction: BLAST (version v2.2.31), ARAGORN (version 1.265) and CRISPRCasTyper (version 1.8.0)
13. AMG validation: VIBRANT (version 1.2.1) and DRAM (version 1.2.0)
14. Sequence alignment and phylogenetic tree construction: MAFFT (version 7.4.9), TrimAl (version 1.5.0) and Fasttree (version 2.1)
15. Recognization of transcriptional promoter: SAPPHIRE (https://sapphire.biw.kuleuven.be/index.php)
16. Protein structural model: Phyre (https://www.sbg.bio.ic.ac.uk/phyre2/)
17. Quality-control of matatranscriptomic reads: Trimmomatic (version 0.35) and SortMeRNA (version 4.3.6)
18. Assembly of matatranscriptomic reads: IDBA_tran (version 1.1.3)

	19. Clustering of matatranscriptomic contigs: Linclust<br>20. Matatranscriptomic reads mapping and quantification: Bowtie2 (version 2.33) and RSEM (version 1.3.3)
Data analysis	See software listed above. And analysis in this project was performed using R version 4.2.2.

For manuscripts utilizing custom algorithms or software that are central to the research but not yet described in published literature, software must be made available to editors and reviewers. We strongly encourage code deposition in a community repository (e.g. GitHub). See the Nature Portfolio guidelines for submitting code & software for further information.

Data

Policy information about availability of data

All manuscripts must include a data availability statement. This statement should provide the following information, where applicable:

- Accession codes, unique identifiers, or web links for publicly available datasets
- A description of any restrictions on data availability
- For clinical datasets or third party data, please ensure that the statement adheres to our policy

The virus sequences generated from contaminated and non-contaminated soil metagenomes in this study have been deposited in the NCBI BioProject database under the accession number PRJNA1196805 and PRJNA1196813, respectively. The soil metagenomic data used in this study are available in the NCBI BioProject database under the accession number PRJNA1253350, PRJNA1253357, PRJNA1253358, PRJNA1253360 and PRJNA1254387. The metatranscriptomic dataset generated from microcosm experiment have been deposited in the NCBI BioProject database under the accession number PRJNA1203377, PRJNA1203296, PRJNA1203294, PRJNA1203300 and PRJNA1203232. The supplementary and source data for this paper are available as Supplementary Data and Source Data files, which have been deposited in the Figshare database [https://doi.org/10.6084/m9.figshare.29924471]. Source data are provided with this paper.

Research involving human participants, their data, or biological material

Policy information about studies with human participants or human data. See also policy information about sex, gender (identity/presentation), and sexual orientation and race, ethnicity and racism.

Reporting on sex and gender	Not relevant to our study.
Reporting on race, ethnicity, or other socially relevant groupings	Not relevant to our study.
Population characteristics	Not relevant to our study.
Recruitment	Not relevant to our study.
Ethics oversight	Not relevant to our study.

Note that full information on the approval of the study protocol must also be provided in the manuscript.

Field-specific reporting

Please select the one below that is the best fit for your research. If you are not sure, read the appropriate sections before making your selection.

☐ Life sciences ☐ Behavioural & social sciences ☒ Ecological, evolutionary & environmental sciences

For a reference copy of the document with all sections, see nature.com/documents/nr-reporting-summary-flat.pdf

Ecological, evolutionary & environmental sciences study design

All studies must disclose on these points even when the disclosure is negative.

Study description	In this study, we explored the effects of viruses on carbon fixation in contaminated soils through spatial investigations at the continental scale combined with experimental verification. Viral and prokaryotic genomes were extensively recovered from contaminated soils in 58 metal mining areas across eastern China. For the first time, 11 types of carbon-fixation auxiliary metabolic genes (AMGs) were identified in the contaminated soils and their functional activities were verified by protein expression. The effects of viral inoculation on the C-fixation function of microbial communities and the changes of soil organic carbon were quantified in the microcosm. Overall, our study provides key insights on the positive effects of viruses on carbon fixation in the contaminated soils, and reinforces the necessary to incorporate viral contributions into assessments of carbon fixation across the ecosystems.
Research sample	50 mining areas were selected according to the national Mineral Resources Development Plan for 2016-2020 and include 16 iron, 18 copper and 16 lead-zinc mines. These three mine types have had the largest provincial funds invested for exploration, and the highest production and consumption of their ores in China. The soil samples for metagenomic sequencing were generated from contaminated (n=323) and non-contaminated (n=86) soils in above mentioned mining areas.<br>The soil for microcosm experiments was from the abandoned land of Dabaoshan mining areas. The viral suspension was derived from soils and used in subsequent viral inoculation experiment. There were 2 treatments established, with five replicates for each sampling time, resulting in 50 culture systems. A total of 50 soil samples were obtained on days 0, 3, 7, 14, and 21 for soil physicochemical analysis and sequencing.

Sampling strategy	At each site, 3–6 soil samples were collected from abandoned mines, tailing areas, downstream contaminated farmlands and non-contaminated sites, making at total of 409 samples. Five soil cores collected from the top 0 - 10 cm were combined into a composite soil sample. All samples were sealed in zipped bags on site and processed immediately upon return to the laboratory. Samples for microbial experiments are collected in sterile tubes and stored at -80 ℃ upon return to the laboratory.<br>The soil in the culture system was divided into three parts. These parts were used for the determination of soil carbon components, RNA extraction and metatranscriptomic sequencing, and RT-qPCR detection, respectively.<br><br>In our study, we used a two-sided t-test to compare the data between the treatment groups (e.g., contaminated soil or non-contaminated soil samples, activated protein or inactivated protein samples and active virus inoculation or inactive virus inoculation samples). The sample size was determined based on practical considerations, including the availability of samples from the experimental setup and the need to achieve sufficient statistical power to detect meaningful differences between the groups.<br><br>We did not perform a formal statistical power analysis to predetermine the sample size prior to the experiment. However, we believe these sample sizes are adequate based on similar studies in the field and our expected effect sizes, The chosen sample sizes provide a balance between statistical power and practical constraints, ensuring reliable and reproducible results.
Data collection	J.L., X.Z. and Z.C. contributed to data collection by sampling contaminated and non-contaminated soils from June 1, 2017 to October 31, 2017 across China (latitude 21°N to 47°N, longitude 99°E to 129°E). . Data were recorded manually using pen and paper during soil sampling.<br>J.L., X.Z. and Z.C. contributed to metagenomic data collection. DNA was extracted from soil samples using Fast DNA®SPIN kit (MP Biomedicals, France). Briefly, about 5 g fresh soil was used for DNA extraction by Fast DNA®SPIN Kit for Soil (MP Biomedicals, France) according to the manufacturer's protocol. The concentration and purity of DNA were evaluated by NanoDrop 2000 (Thermo Fisher Scientific, USA). Finally, 409 purified DNA were chosen for metagenomic sequencing on the Illumina NovaSeq platform (350 bp insert size, paired-end 150 bp) by Novogene (Beijing, China). Sequencing reads were processed for quality control, contig assembly, viral sequence identification and ecological analyses.<br>J.L. contributed to metatranscriptomic data collection. RNA was extracted from 2 g soil samples in microcosm incubation using RNeasy PowerSoil Total RNA Kit on days 0, 3, 7, 14, and 21, which purity and concentration were determined by NanoDrop 2000 (Thermo Fisher Scientific, USA). rRNA transcripts were eliminated by ALFA-SEQ rRNA depletion kit (for bacterial). After the library was qualified, Illumina HiSeq platform was used for paired-end sequencing. Sequencing reads were processed for quality control, contig assembly and ecological analyses.<br>The contents of soil organic/inorganic carbon, 13C labeled organic carbon and carbon dioxide were measured using elemental analyzer (Vario EL cube, Germany), isotope ratio mass spectrometers (DELTA V Advantage, USA) and gas chromatograph (GC-2014, China), respectively. Data were recorded and directly entered into computer-based analysis software.
Timing and spatial scale	A continent-scale survey of metal mining areas in eastern China was conducted from June to October 2017 (latitude 21°N to 47°N, longitude 99°E to 129°E).
Data exclusions	No data have been excluded.
Reproducibility	Spatial investigations at the continental scale were conducted with 3-6 biological replicates.<br>Enzyme activities were successfully confirmed with three replicates using distinct samples.<br>Viral inoculation experiment was conducted with five biological replicates.
Randomization	It is not relevant to our studies because we conducted random sampling across China and the samples were not allocated into groups. The number of replications equals the number of sample in our case.
Blinding	During data analysis, blinding was conducted by only taking the sample ID into account and the sampling location was not involved.

Did the study involve field work? ☒ Yes ☐ No

Field work, collection and transport

Field conditions	The climatic conditions of the sampling sites varied considerably, with mean annual precipitation ranging from 230 to 1942 mm.
Location	The sampling sites covered latitude from 21°N to 47°N, and longitude from 99°E to 129°E
Access & import/export	Import/export is not relevant to our study.
Disturbance	Only topsoil (i.e., 0-10 cm in depth) wascollected. No disturbances were caused by this study.

Reporting for specific materials, systems and methods

We require information from authors about some types of materials, experimental systems and methods used in many studies. Here, indicate whether each material, system or method listed is relevant to your study. If you are not sure if a list item applies to your research, read the appropriate section before selecting a response.

Materials & experimental systems

n/a	Involved in the study
☒	☐ Antibodies
☒	☐ Eukaryotic cell lines
☒	☐ Palaeontology and archaeology
☒	☐ Animals and other organisms
☒	☐ Clinical data
☒	☐ Dual use research of concern
☒	☐ Plants

Methods

n/a	Involved in the study
☒	☐ ChIP-seq
☒	☐ Flow cytometry
☒	☐ MRI-based neuroimaging

Plants

Seed stocks	Not relevant to our study.
Novel plant genotypes	Not relevant to our study.
Authentication	Not relevant to our study.
